# Advancements, challenges, and future prospects of smart grid technology in India

**DOI:** 10.3389/frai.2024.1475604

**Published:** 2024-11-11

**Authors:** Archana Talhar Belge, Shiwani Gupta, Sujata Alegavi, Vidyadhari Singh, Kshama Shukla

**Affiliations:** ^1^Department of Mechanical and Mechatronics Engineering, Thakur College of Engineering and Technology, Mumbai, India; ^2^Department of B.Tech. AI&ML, Thakur College of Engineering and Technology, Mumbai, India; ^3^Department of B.Tech. IoT, Thakur College of Engineering and Technology, Mumbai, India; ^4^Department of Computer Science and Engineering, Thakur College of Engineering and Technology, Mumbai, India

**Keywords:** smart grid, generation, distribution, consumption, communication, digital technology, renewable sources

## 1 Introduction to Indian electricity grid

The Indian energy grid, with a capacity of 250 GW, comprises five regional grids and operates on a single frequency. It consists of three functional blocks: Generation, Transmission, and Distribution. Each grid's operation is overseen by the Regional Load Despatch Center (RLDC), which works closely with the State Load Despatch Center (SLDC) in each of its constituent states. The National Load Despatch Center (NLDC) oversees coordination between the five RLDCs and interregional and international exchange. Traditional grids in India face issues like longer fault clearing times, lack of automatic fault healing, voltage sag, voltage swell, and power theft. Converting the current grid into a smart grid could reduce these issues (Central Electricity Authority, 2024).

Smart grid, which delivers electricity to consumers through two-way digital communication, is built on digital technologies. This system facilitates communication, analysis, control, and monitoring throughout the energy supply chain to improve efficiency, reduce energy prices and consumption, and raise transparency and dependability of the chain (Sharma and Kamth, 2018; Pei et al., 2019; Shamim and Rihan, 2017; Tetteh and Awodele, 2019).

The development of a smart grid is necessary because it can have: (a) Increased utilization of renewable energy sources, (b) Broad and efficient generation-to-consumer communication overlap, (c) Utilizing cutting-edge sensors and rapid control, (d) More operational effectiveness, (e) Enhanced ability to withstand attacks and natural catastrophes, (f) Fast power restoration and automated metering, and (g) Increased consumer involvement. In papers (Vyas et al., 2019; Colak et al., 2020; Sreedevi et al., 2020; Zhang et al., 2020; Zhou et al., 2020), the basic requirements of the smart grid are discussed in detail like, Grid Reliability and Resilience, Integration of Renewable Energy, Energy Storage Systems, Demand Response Management, Electric Vehicle (EV) Integration, and Cybersecurity etc.

Smart grids are advanced energy management systems that use advanced technologies to detect and prevent power outages, optimize energy efficiency, and support sustainability by integrating renewable energy sources. They prioritize security through cybersecurity, offer flexibility through energy storage systems, and ensure interoperability through standardized communication protocols. Real-time monitoring systems provide real-time visibility into grid operations and energy demand. These systems are cost-effective, resilient, and enable utilities to make informed decisions. The details are illustrated in Supplementary Figure 2 (Colak et al., 2020).

In an effort to guarantee the efficient operation of a smart grid, several key requirements must be met. These include precisely estimating the amount of energy produced and consumed, keeping grid variables like frequency, voltage, and current constant, and balancing the amount of power produced and consumed during peak and off-peak hours. Numerous advantageous aspects of smart grid directly benefit consumers (Colak et al., 2020; Sreedevi et al., 2020; Zhang et al., 2020; Zhou et al., 2020; Sharma et al., 2017; Routray et al., 2021; Tianqi et al., 2021; Zhang et al., 2021; National Smart Grid Mission Ministry of Power, Government of India, 2024).

Smart grids enable real-time tracking of energy use and grid functioning, allowing fault detection and optimization. Automated outage management reduces power disruptions, improving grid reliability and customer satisfaction. Dynamic pricing schemes encourage users to shift energy consumption during off-peak hours, lowering peak demand and strain on the grid. Web portals and mobile apps enhance efficiency and convenience. Smart grid technology uses IT and ICT infrastructure, including Advanced Metering Infrastructure (AMI), peak load management, Power Quality Management, and Outage Management System (OMS) to manage infrastructure breakdowns.

Microgrids integrate distributed energy resources and interconnected loads, providing reliable and resilient electric power. Distributed Generation technologies improve customer interaction flexibility, energy usage, demand management, loss reduction, transaction management, and pricing and billing systems, fostering a more efficient and sustainable energy ecosystem (National Smart Grid Mission Ministry of Power, Government of India, 2024). An analysis of the development of smart grids in five developed nations—Australia, Canada, China, Germany, India, and Japan—is presented by the author (Sharma et al., 2017). They analyse China's smart grid industry and its development chain (He and Zou, 2020). The paper also discusses the China-Pakistan Economic Corridor (CPEC) initiative and discusses joint pilot projects (Ul-Haq et al., 2021). The article highlights advancements in new energy development in China, particularly in the northwest region, and introduces the concept of a highly resilient transmission power grid to support high-proportion new energy grid growth (Niu et al., 2023).

## 2 Smart grid pilot project developments in India, China, United States, and European Union

The Ministry of Power has sanctioned smart grid pilot projects under the Integrated Power Development Scheme (IPDS), which are discussed below (National Smart Grid Mission Ministry of Power, Government of India, 2024):

1. Ajmer Vidyut Vitran Nigam Ltd. (AVVNL), Ajmer: A pilot test is underway for 1,000 consumers to implement functionality as Advanced Metering Infrastructure (AMI), utilizing a hybrid setup that integrates smart and traditional meters, assessing their feasibility and effectiveness within the same AMI framework. Better energy auditing and a decrease in AT&C losses are two advantages.

2. Assam Power Distribution Company Limited (APDCL), Assam: A pilot project with 15,000 users and 90 MUs of input energy capacity which is ongoing. It uses SCADA/DMS infrastructure for future Smart Grid development and integrates solar power and DG backup to improve energy mix and resilience. The deployment of Advanced Metering Infrastructure, Distributed Generation, Peak Load Management, and Outage Management has enhanced energy availability, spurred revenue growth, and decreased AT&C losses.

3. Chamundeshwari Electricity Supply Corporation Limited (CESC), Mysore: The project includes 512 irrigation pump sets, 14 feeders, 473 transformers, 21,824 consumers, and can handle 151.89 MU of energy input. It includes agricultural demand management, consumer portals, performance-based management information system, and data analytics. Peak load usage, billing expenses, and distribution losses have all decreased as a result of deployment of Advanced Metering Infrastructure, Distributed Generation, Peak Load Management, and Outage Management.

4. Himachal Pradesh State Electricity Board Limited (HPSEB), Himachal Pradesh: The project aims to enhance power quality management by installing advanced meters at 1,251 high-tension consumers, enabling swift identification and corrective actions to facilitate consumer decision-making. The functionality implemented is 80% similar to CESE. The benefits of same are shifting peak load, reduction in penalties and outages.

5. Electricity Department-Puducherry (PED), Puducherry: The project, part of the RAPDRP Scheme, aims to enhance IT infrastructure and system for 34,000 domestic users, including a Common Meter Data Management System for data aggregation.

6. Tripura State Electricity Corporation Ltd (TSECL), Tripura: A pilot project, part of the RAPDRP Scheme, is being implemented to enhance IT infrastructure and strengthen the energy system. For effective energy management, Tariff schemes for Net Metering and Time of Use models are essential components. The implemented functionality, identical to AVVNL, offers benefits such as reduced distribution losses, reduced billing costs, and increased revenue collection efficiency.

7. Telangana State Southern Power Distribution Company Limited (TSSPDCL), Telangana: A pilot project targeting 45,290 consumers is underway for IT infrastructure development and system enhancement under the RAPDRP Scheme. The project explores innovative pricing mechanisms to optimize energy management and consumption patterns. The implemented functionality and benefits are identical to APDCL, resulting in lower AT&C losses and peak load usage, and billing cost reduction.

8. Uttar Haryana Bijli Vitran Nigam Limited (UHBVN), Haryana: A pilot project, part of the RAPDRP Scheme, is being implemented to enhance IT infrastructure and energy system efficiency for 11,000 consumers with an annual energy consumption of 70 MMU. The functionality implemented is 100% similar to APDCL and benefits are similar to TSSPDCL.

9. Uttar Gujarat Vij Company Limited (UGVCL), Gujarat: The pilot project in Naroda aims to improve energy supply efficiency and reliability for 22,230 consumers by improving utility-level functions such as asset management, peak power management, load forecasting, and outage management. The implemented functionality, identical to CESE, offers numerous benefits including reduced AT&C losses, peak power purchase costs, transformer failure rates, outages, meter reading costs, and payment collection costs.

10. West Bengal State Electricity Distribution Company Limited (WBSEDCL), West Bengal: The proposed project area, which serves 5,265 consumers, has an electrical infrastructure consisting of two 11 kV feeders and 46 distribution transformers, consuming approximately 7.46 million units annually. The implemented functionality benefits are very much similar to CESE.

11. IIT Kanpur: The project aims to develop an R&D platform and Smart City prototype, showcasing advanced smart distribution systems and urban infrastructure, including automation upgrades, smart home integration, and grid-connected solar PV installation. This project includes substation automation, rooftop solar PV integration, smart houses, IT infrastructure, renewable integration, advanced metering infrastructure, and smart home management system.

12. Smart Grid Knowledge Center (SGKC), Manesar: The Smart Grid Knowledge Center will provide technical assistance to NSGM, enhancing workforce development, capacity enhancement, and outreach initiatives, fostering a supportive ecosystem for smart grid advancement. The project includes AMI, OMS, Micro Grid/Distributed Generation, EV charging, Home Energy Management Center, Cyber Security & Training Infrastructure, and a laboratory platform for Smart Grid functionalities. The state-of-art methodologies play crucial role in advanced utilization of smart grid technology in US, EU, and China and are noted below (Sospiro et al., 2021):

Reviewing the overall purpose and goals, identifying functions, evaluating project characteristics, mapping each function onto a standardized set of benefits, setting baselines, gathering data, quantifying benefits, monetizing benefits, and estimating costs are all part of the state-of-art methodologies for the growth of smart grid technology. While some technologies, such as information and communication technology (ICT), distributed generation (DG), energy storage system (ESS), and electric vehicle (EV) infrastructure, have flourished, others, such as Wide Area Management Systems (WAMS), Distribution Automation (DA), Advanced Metering Infrastructure (AMI), and Customer Technology (CT), and Smart Electricity Market (SEM) are not yet explored. The implementation of WAMS in China and US is 33%, while in EU it is only 25%. The maximum implementation of Advanced Metering Infrastructure is 84% in China, 50% in US, and 80% in EU. The minimum implementation for Customer Technology is 13% in China, 28% in the US, and 13% in the EU.

## 3 Challenges faced in smart grid implementation

Numerous issues with smart grid technologies need to be resolved (Sharma et al., 2017; Routray et al., 2021; Tianqi et al., 2021). Smart grids face numerous challenges such as demand, power transmission, forecasting, cybersecurity risks, infrastructure upgrades, data management, regulatory frameworks, public acceptance, workforce training, grid resilience, scalability, standardization, consumer engagement, renewable energy integration, grid modernization, and energy storage stability.

After the execution of smart grid projects in India, some of the major challenges faced are (Kappagantu and Daniel, 2018):

The current grid infrastructure is inadequate for clean energy and distributed generation, presenting challenges in design, installation, operation, and maintenance. Cyber security is crucial to prevent security breaches. Smart grids integrate renewable energy sources for large-scale power generation but require storage solutions due to intermittent and renewable energy production. Common options include batteries, flywheels, thermal storage, and hydrogen storage, but face challenges due to potential raw material scarcity. Effective data management is necessary for smart grids, but communication issues persist with unstandardized technologies and vulnerable wired communication methods. Socio-economic challenges, such as high capital investment, stakeholder engagement, system operation, privacy concerns, fear of obsolescence, increased electricity charge, new tariffs, and radiofrequency signal and health issues, can hinder the relevance of smart grids.

To prevent load shedding, energy storage devices must be used, and power transmission must match characteristics at sending and receiving ends. Technical challenges include large dispersed nodes, interoperability, and data management. Public acceptance, workforce training, and scalability are essential for successful smart grid implementation. Standardization and consumer engagement are also crucial for grid stability and reliability.

## 4 Possibilities for integration of smart grid through inclusion of AI

AI is revolutionizing India's smart grid infrastructure by predicting electricity demand, enhancing load distribution, and reducing blackout risks. AI can monitor grid components' health in real-time, identifying faults before they cause significant problems, leading to cost savings. It can control the unpredictability of renewable energy sources', including solar and wind power, providing a reliable energy supply. AI systems can better identify and respond to cyberthreats, protecting the grid from disruptions. AI-driven demand-side management programs encourage consumers to reduce energy usage during peak times, while smart appliances and home automation systems optimize energy consumption. AI can also optimize grid operations by balancing supply and demand in real-time and implementing dynamic pricing models. It can also assist in disaster management by predicting and mitigating natural disaster impacts.

## 5 Conclusion

This paper investigates the current state of Indian electricity grid and the government's plans to transform it into a smart grid. The partial focus given on the happening related to SG technologies in other countries like China, US, and EU. It outlines the essential requirements, features, challenges, and key constituents of smart grid system. The effectiveness of smart grid implementation is demonstrated through various pilot projects initiated by Indian government.

The discussion concludes that smart grid technology provides numerous benefits, including reduced transmission and distribution (T&D) losses; peak load control; enhanced asset management; reduced power purchase prices; enhanced service quality and dependability; enhanced grid visibility and self-healing capabilities; and alternatives like Time-of-Use (ToU) tariffs, demand response schemes, and net metering. These improvements lead to higher customer satisfaction and financially stable utilities. The paper also presents government-implemented pilot projects supporting smart grid deployment.

Moreover, the integration of AI into smart grids offers several benefits, including improved data privacy and security, significant investment in infrastructure upgrades, development of appropriate regulatory and policy frameworks, and establishment of a skilled workforce for developing, deploying, and maintaining AI systems.
