# Supplementary material for: Advancements, challenges, and future prospects of smart grid technology in India
Source: Front Artif Intell. 2024 Nov 11;7:1475604. doi: 10.3389/frai.2024.1475604 (PMC11586363; doi:10.3389/frai.2024.1475604)
Supplement: Supplementary file 1 [file Data_Sheet_1.docx]

Supplementary Material


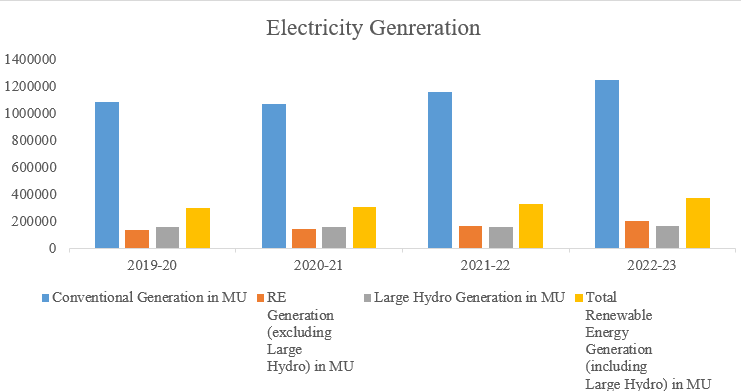


Fig. 1 All India electricity generation from 2019 to 2023.


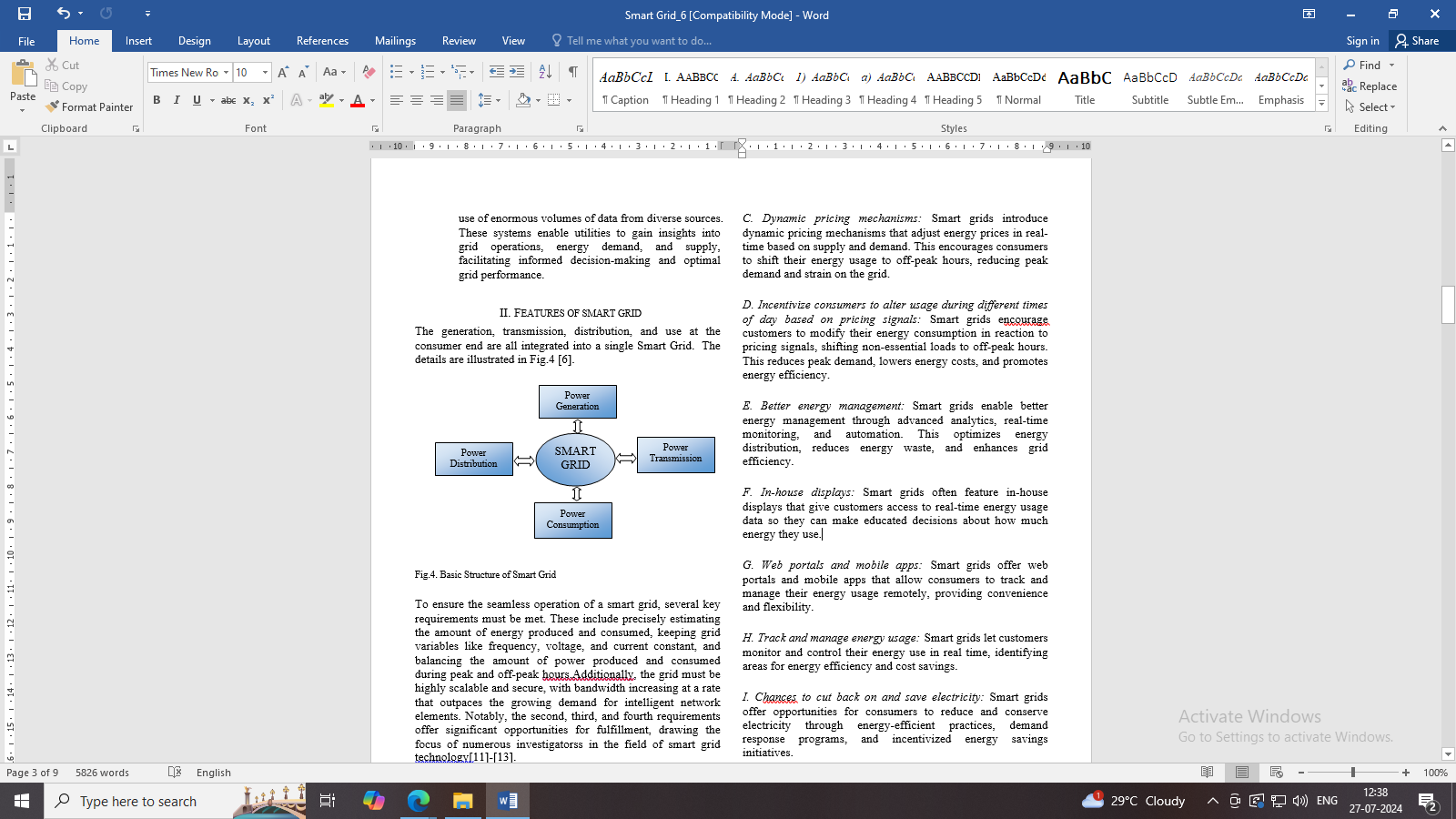


Fig.2. Basic Structure of Smart Grid
